# RETRACTED: Shu et al. Using Stable Isotope Techniques to Analyze the Trophic Relationship between Argentine Hake (*Merluccius hubbsi*) and Anisakidae. *Biology* 2024, *13*, 515

**DOI:** 10.3390/biology14070860

**Published:** 2025-07-16

**Authors:** Yue Shu, Feiyu Wu, Zhou Fang

**Affiliations:** 1 College of Marine Living Resource Sciences and Management, Shanghai Ocean University, Shanghai 201306, China; shuyue0622@163.com (Y.S.); 15935148312@163.com (F.W.); 2 National Engineering Research Center for Oceanic Fisheries, Shanghai Ocean University, Shanghai 201306, China; 3 Key Laboratory of Sustainable Exploitation of Oceanic Fisheries Resources, Ministry of Education, Shanghai Ocean University, Shanghai 201306, China; 4 Key Laboratory of Oceanic Fisheries Exploration, Ministry of Agriculture and Rural Affairs, Shanghai 201306, China; 5 Scientific Observing and Experimental Station of Oceanic Fishery Resources, Ministry of Agriculture and Rural Affairs, Shanghai 201306, China

The journal *Biology* retracts the article titled “Using Stable Isotope Techniques to Analyze the Trophic Relationship between Argentine Hake (*Merluccius hubbsi*) and Anisakidae” [1], cited above.

Following the publication, concerns were brought to the attention of the Editorial Office regarding the presence of unverifiable sources in the cited literature and the scientific accuracy of the findings presented in this study [1].

Adhering to our complaint’s procedure, the Editorial Office and Editorial Board conducted an investigation that confirmed the presence of several unverifiable citations as well as a number of significant errors and limitations in the study. As a result, the Editorial Board has lost confidence in the reliability of the conclusions and decided to retract this publication [1], as per MDPI’s retraction policy (https://www.mdpi.com/ethics#_bookmark30).

This retraction was approved by the Editor-in-Chief of the *Biology* journal.

The authors disagreed with this retraction.
